# Changes in Acute-Phase Proteins in Plasma during the Periparturient Period of Dairy Goats

**DOI:** 10.3390/vetsci8120311

**Published:** 2021-12-08

**Authors:** Fangyuan Zeng, Bingyu Shen, Yang Yuan, Yezi Kong, Panpan Tan, Yan Huang, Yaoquan Liu, Siqi Liu, Baoyu Zhao, Jianguo Wang

**Affiliations:** College of Veterinary Medicine, Northwest A&F University, Xianyang 712100, China; zengfy0106@163.com (F.Z.); shenby@nwafu.edu.cn (B.S.); yuanyang0115@163.com (Y.Y.); kongyezi0207@163.com (Y.K.); Tanpp@nwsuaf.edu.cn (P.T.); hy040016@163.com (Y.H.); liuyq0703@163.com (Y.L.); liusiqi647777@163.com (S.L.); zhaobaoyu12005@163.com (B.Z.)

**Keywords:** acute-phase proteins, dairy goats, peripartum

## Abstract

The present study was conducted regarding four acute-phase proteins (APPs) including C-reactive protein (CRP), ceruloplasmin (CP), serum amyloid A (SAA), and haptoglobin (HP) in dairy goats during the periparturient period. The aim of this study was to detect the changes in APPs in plasma during the periparturient period of healthy dairy goats. Guanzhong dairy goats with no other symptoms (*n* = 15) were selected on the basis of their blood calcium (Ca) and β-hydroxybutyrate (BHBA) concentration. The plasma was collected once a week for ±3 weeks delivery. The concentrations of the four APPs mentioned above were determined using goat-specific ELISA kits. The results showed the CRP level in plasma decreased from 3 weeks to 1 week antepartum and increased later until 1 week postpartum and then decreased to a similar level with antepartum between 1 and 3 weeks postpartum. The content of CP showed a decline in 3 weeks before parturition and an upward trend between 1 week antepartum and 3 weeks postpartum. The SAA concentration decreased from 3 weeks antepartum to 2 weeks postpartum and rebounded later. The level of HP decreased during 3 weeks before parturition and increased until 1 week postpartum, then reached a stable value. Clear variation range and rules of APPs contribute to perinatal health monitoring of dairy goats.

## 1. Introduction

The periparturient period is critical for dairy goats. In this period, the dry matter intake (DMI) cannot compete with the required demands for fetal growth and milk production [1,2]. Hence, dairy goats are vulnerable to suffering from negative energy balance (NEB) and metabolic disorders during this period, which further progress to some severe diseases such as ketosis, laminitis, mastitis, metritis, and milk fever [3,4]. Due to the lack of early diagnosis, these diseases might have risks of developing more severe and irretrievable outcomes.

APPs are mostly synthesized by the liver in the acute phase. When an organism is under injury, infection, and inflammation, the activated monocytes and injury tissue can secret pro-inflammatory cytokines, such as interleukin-1 (IL-1), interleukin-6 (IL-6), and tumor necrosis factor (TNF-α), which can induce the secretion of APPs [5]. CRP, CP, SAA, and HP are several representative APPs. The concentrations of the four APPs increased sharply when inflammation emerged and decreased rapidly with diseases fading; thus, they are widely known as non-specific markers of inflammation.

The detection of APPs is convenient, efficient, and sensitive. It has been widely used in clinical diagnosis on humans and small animals. However, the application was limited due to the lack of theoretical basis and data support in small ruminants. Samimi et al. reported the changes in APPs in three different physiological times [6]. However, the information on the changes in APPs during the periparturient period is still insufficient. The present study was conducted to monitor changes in four APPs in healthy goats during the periparturient period.

## 2. Materials and Methods

The Northwest A&F University Institutional Animal Care and Use Committee approved all procedures involving animals in this study. A commercial dairy goat farm located in Shaanxi Province of China was selected to participate in the study.

### 2.1. Selection of Goats

**Figure 1:**
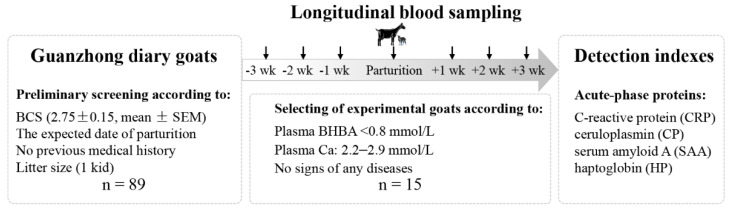
A two-step strategy to screen experimental animals.

The study design is schematically presented in Figure 1. A two-step strategy was used to screen experimental animals as follows: (i) a total of 96 non-lactating primiparous goats in late pregnancy were randomly selected from 2305 Guanzhong dairy goats according to body condition score (BCS) (2.75 ± 0.15, mean ± SEM), the expected date of parturition (within the first week of February), and no previous medical history. Furthermore, 89 goats were selected for their same litter size (1 kid). All goats were raised under the same environmental and managerial conditions. The same diets were fed during the experiment, and the nutrient requirements of dairy goats during the periparturient period could be satisfied. The diets were referred to the Nutrient Requirements of Small Ruminants (National Research Council, 2007), including alfalfa hay, corn, wheat bran, soybean meal, wheat straw, corn silage, corn germ meal, cottonseed meal, and minerals. The diets and water were provided twice a day at 7:30 and 15:30 ad libitum throughout the entire trial period. (ii) The blood samples (10 mL) were collected from the jugular vein of each goat once a week for ±3 weeks delivery. In antepartum, the blood samples were collected before feeding in the morning. On the delivery day, the blood samples were obtained within 24 h after delivery. In postpartum, the collection of blood samples was conducted in the period between the milking operations and feeding in the morning. All blood samples were collected in 10 mL vacuum blood collection tubes with heparin sodium (Becton-Dickinson, Franklin Lakes, NJ, USA). The blood samples were centrifuged at 2000× *g* for 10 min, and the separated supernatant plasma was collected and stored at −80 °C properly for later analyses. With the criteria of 2.2–2.9 mmol/L Ca and BHBA <0.8 mmol/L according to the blood analyses, 53 goats were considered as the healthy group. In total, 15 goats who showed no signs of any diseases during the trial period were eventually involved as the object of the study. All the animals were released after the study.

### 2.2. Plasma Analyses

The concentration of BHBA in plasma was analyzed by commercial kits (kit no. RB1007, enzymatic method, Randox laboratories, Crumlin, UK), and Ca (kit no. CA590, Arsenazo Ⅲ method) was analyzed by Hitachi auto-analyzer (Hitachi High-Technologies Corporation, Tokyo, Japan). The concentrations of CRP (MM-75014O1), CP (MM-35263O1), SAA (MM-1291O1), and HP(MM-35246O1) in plasma samples were measured by double antibody sandwich method with goat-specific ELISA kits (Meimian Biotechnology, Yancheng, Jiangsu, China) and the Bio-Rad 680 microplate reader (Bio-Rad, Hercules, CA, USA). The optical density (OD) values were determined at a wavelength of 450 nm, and the results were calculated by comparing the OD of samples to the standard curve.

### 2.3. Statistical Analyses

The obtained data were statistically analyzed by using GraphPad Prism 7.0 (GraphPad Software Inc., La Jolla, CA, USA). The changes in the APP concentrations in plasma were analyzed using repeated measurements ANOVA, followed by Tukey’s multiple comparison tests. Repeated measures on each goat were considered (repeated factor: time during the periparturient period). The results are expressed as mean ± standard error of the mean (SEM).

## 3. Results

**Figure 2:**
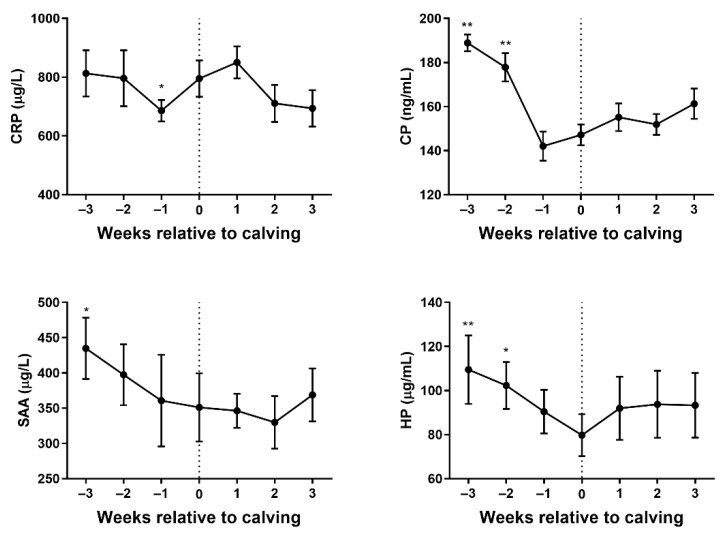
Changes in plasma concentrations of C-reactive protein (CRP), ceruloplasmin (CP), serum amyloid A (SAA), and haptoglobin (HP) in dairy goats at ±3 weeks delivery. Statistical significance was indicated at * *p* < 0.05 and ** *p* < 0.01 in comparison with the delivery day.

The variation in concentrations of CRP, CP, SAA, and HP in plasma samples during the periparturient for 15 healthy goats are presented in Figure 2, respectively. The plasma’s CRP level decreased from 3 weeks prior to parturition and reached a minimum value at 1 week prior to parturition. The plasma’s CRP concentration had a significant difference between 1 week prior to parturition and the parturition day (*p* < 0.05). The plasma’s CRP concentration then increased until 1 week postpartum, followed by a sharp decrease to a similar level with antepartum between 1 and 3 weeks postpartum. The content of CP showed a decline in 3 weeks before parturition. The plasma’s CP concentration had a significant difference at both 3 and 2 weeks before parturition, compared with parturition day (*p* < 0.01). The plasma’s CP concentration showed an upward tendency between 1 week antepartum and 3 weeks postpartum, and the least value was observed at 1 week before parturition. The SAA concentration was decreased gradually from 3 weeks antepartum to 2 weeks postpartum and rebounded later. The difference between 3 weeks antepartum and the parturition day (*p* < 0.05) was significant. The level of HP decreased gradually during 3 weeks before parturition and increased until 1 week postpartum, then reached a stable value. It was observed that the plasma’s HP concentration had a significant difference between 3 weeks before parturition (*p* < 0.01), 2 weeks before parturition (*p* < 0.05), and the parturition day.

## 4. Discussion

As a sensitive test, prevalent and in-depth studies about APPs also have been conducted on animals. In dairy cows, APPs have been identified as markers of inflammation. It has been reported that the concentrations of SAA and HP were higher in the serum and milk of mastitis cows than in healthy cows [7]. Lame cattle exhibit high levels of APPs including CRP, HP, and SAA, as compared with non-lame cattle [8]. The changes in SAA, HP, and CP concentrations in plasma were significant relative to both uterine bacterial contamination in cattle after calving and uterine involution [9]. On the other hand, SAA and HP can be used to discriminate between acute and chronic inflammatory conditions. It is reported that the concentrations of SAA and HP in cattle with acute inflammation were significantly higher than in cattle with chronic inflammation [10]. Furthermore, APPs have been linked to chronic inflammation induced by metabolic diseases [11].

The metabolism of dairy goats during the periparturient period is unstable on account of the fetus’s growth and lactation, which leads to a high risk of diseases. Several studies have shown inflammation during the periparturient period has been affiliated with disorder metabolism [12,13]. In addition, the plasma concentrations of sex hormones in sheep change dramatically as the animal approaches parturition [14]. It was reported the fluctuation of sex hormones negatively affects the function of the immune system, and it was confirmed that mild immunosuppression existed in diary sheep during the periparturient period [15]. Hence, we supposed that the undulation of sex hormones impacts the immune system in dairy goats, which further influences the level of APPs.

CRP can recognize damaged cells as well as their products, and it can repair the nidus via activating complement. CRP is one of the most rapid reacting APPs, it is induced primarily by IL-1, and the plasma’s CRP concentration increases within 4 h after stimulation [16,17]. Lee WC et al. reported that CRP can evaluate the general health status of a herd [18]. The plasma’s CRP concentration decreased from 3 weeks to 1 week prior to parturition. The downward trend of APPs before delivery may be induced by the drastic changes in hormones and immunosuppression. There was an increase in the plasma’s CRP concentration on the delivery day and 1 week after delivery, indicating that mild inflammation existed in this period. IL-1 plays a role in increasing prostaglandin (PG) synthesis. A high level of PG is one crucial factor in labor onset and the increase in CRP may be due to the rise of PG. From 1 week after delivery, the CRP level decreased and reached the same level 1 week before delivery, which may be attributed to the alleviation of inflammation or the regulation of the hormones. The plasma’s CP concentration dropped rapidly at 1 week antepartum in our study. Okumura M reported that the CP level decreased before and after delivery in mare, which accord closely with our results [19]. The content of CP increased in the middle and later period of acute inflammation. We can infer that the increased concentration of CP in dairy goats between 1 week antepartum and 3 weeks postpartum may be influenced by the variation in sex hormones and inflammation. SAA is characterized by high sensitivity, and it may also be increased in the condition of inflammation without clinical signs. It is suggested that SAA can reflect systemic inflammatory disease in screening dairy cow herds [20]. The decreased SAA concentration reached the minimum level at 2 weeks after delivery, followed by a slight increase, according to our research. The SAA concentration during the postpartum period was at a low level in comparison with 3 weeks antepartum. However, the concentration of SAA did not show a distinct change during the parturition period. SAA has been proposed not to be influenced by sex hormones [21]. Thus, the changes in the SAA level may just reflect the condition of inflammation and immunosuppression. HP is induced primarily by IL-6, and it could combine hemoglobin released by damaged erythrocytes to alleviate the harm of free iron. The time point of the increase in the HP concentration might be later, and the level can last for a long time [22]. In the present study, the plasma’s HP concentration began to increase at the delivery day and kept a level for 3 weeks. It is worth noting that the plasma’s HP concentration was at a low level after delivery, indicating the dairy goats might have suffered from immunosuppression in this period.

## Data Availability

The raw data supporting the conclusions of this article will be made available by the authors, without undue reservation.
